# Genetic Modifiers of Parkinson's Disease: A Case–Control Study

**DOI:** 10.1002/acn3.70176

**Published:** 2025-09-10

**Authors:** Matthew J. Kmiecik, Michael V. Holmes, Pierre Fontanillas, Giulietta M. Riboldi, Ruth B. Schneider, Jingchunzi Shi, Anna Guan, Susana Tat, Steven Micheletti, Keaton Stagaman, Josh Gottesman, David A. Hinds, Joyce Y. Tung, 23andMe Research Team, Stella Aslibekyan, Lucy Norcliffe‐Kaufmann

**Affiliations:** ^1^ 23andMe, Inc. Sunnyvale California USA; ^2^ The Marlene and Paolo Fresco Institute for Parkinson's and Movement Disorders NYU Grossman School of Medicine New York New York USA; ^3^ University of Rochester Medical Center Rochester New York USA; ^4^ The Michael J. Fox Foundation for Parkinson's Research New York New York USA

**Keywords:** *GBA1*, *LRRK2*, neurodegeneration, Parkinson's disease, polygenic risk scores

## Abstract

**Objective:**

To examine the associations of *LRRK2* p.G2019S, *GBA1* p.N409S, polygenic risk scores (PRS), and *APOE* E4 on PD penetrance, risk, and symptoms.

**Methods:**

We conducted a US‐based observational case–control study using data from the 23andMe Inc. and Fox Insight Genetic Substudy (FIGS) databases. The total cohort included 7,586,842 participants (*n* = 35,163 PD); 8791 *LRRK2* p.G2019S carriers (565 with PD), 37,427 *GBA1* p.N409S carriers (524 with PD), 244 dual *LRRK2*/GBA1 carriers (37 with PD), and 7.5 million noncarriers (34,037 with PD). PRS was calculated from the most recently published European genome‐wide association study. Survival models estimated the cumulative incidence of PD. Logistic regressions estimated the relative odds of reporting motor and non‐motor symptoms according to genetic exposure.

**Results:**

By the age of 80 years, the cumulative incidence of PD was 30% for dual carriers, 24% for *LRRK2* p.G2019S carriers, 4% for *GBA1* p.N409S carriers, and 2% for noncarriers. Higher PRS was associated with increased penetrance of the variants and earlier time to PD diagnosis. *GBA1* p.N409S PD was associated with the highest burden of non‐motor symptoms, including REM sleep behavior disorder and cognitive/memory deficits, and *LRRK2* p.G2019S with the lowest. *APOE* E4 dosage was associated with greater odds of reporting hallucinations and cognitive impairment in addition to carrier status.

**Interpretation:**

Our findings support the use of genetic screening to enrich candidate selection for neuroprotective trials and better define outcome measures based on genetics.

The progression of Parkinson disease (PD) is currently untreatable. For inherited forms of PD, it is possible to intervene before pathological changes become manifest clinical symptoms at a time when disease‐modifying therapies would be expected to have more impact [1, 2].

The most common genetic variants associated with PD risk are located within the *LRRK2* (2% of PD cases) and *GBA1* (10%) genes [3, 4, 5, 6, 7]. *GBA1* encodes for glucocerebrosidase (GCase), a lysosomal enzyme. GCase activity is reduced in the presence of the p.N409S missense variant [rs76763715; *GBA1* NM_000157.4:c.1226A>G (p.Asn409Ser); previously named p.N370S]. *LRRK2* encodes for a kinase and GTPase complex, and the common p.G2019S missense variant results in a gain of function in the kinase domain [rs34637584; *LRRK2* NM_198578.4:c.6055G>A (p.Gly2019Ser)]. Despite the growing interest in recruiting at‐risk genetic cohorts, several hurdles remain to design a PD prevention study. Neither the p.N409S or p.G2019S variants are fully penetrant (i.e., not all carriers will develop PD); PD is associated with a long preclinical phase, and it is not known which key features can be used to predict progression from mild symptoms to manifest disease in genetic forms [8]. Without the ability to select an enriched subgroup of carriers likely to phenoconvert, there is no way to shorten the period of assessment or reduce the number of participants required in an early intervention clinical trial.

Genetic predisposition appears to be important in the pathology of PD. At least one‐third of *LRRK2* carriers may have a neurodegenerative process that is independent of α‐synuclein aggregation in the substantia nigra, which is the pathologic hallmark of PD [9]. Hundreds of additional genetic regions are linked to PD susceptibility [10, 11, 12]. Polygenic risk scores (PRS) are driven by the count and weight of risk allele frequencies, and individuals at the top PRS quantiles are at particularly high risk of developing PD at an earlier age [13, 14, 15].

The challenge remains to improve the precision of endpoints and to determine when to select motor versus cognitive decline as the primary outcome of interest. Although neurons in the substantia nigra are particularly susceptible to α‐synuclein‐mediated neurodegeneration, other regions of the brainstem, cortex, and periphery are also impacted. *GBA1* PD carries more risk of dementia [16], whereas *LRRK2* PD appears to have a more restricted pattern of neurodegeneration and reduced risk of dementia [15, 17]. The link between the apolipoprotein E (*APOE*) E4 allele and cortical neurodegeneration is well known in Alzheimer's dementia [18, 19] and the pathological overlap between PD and Alzheimer's is well established, with tau and α‐synuclein deposition often found in combination [20]. More recently, it has emerged that *APOE* E4 positive carrier status may also be linked to dementia with Lewy bodies (DLB) [21] and PD dementia [22]; both show α‐synuclein‐containing Lewy bodies in the cortex [23, 24]. Whether *APOE* E4 carrier status poses a similar risk of dementia in *LRRK2* and/or *GBA1* PD is unknown.

We performed analyses using an aggregated cohort of 35,163 PD cases and 7,506,343 non‐PD cases to investigate genotype/phenotype relationships in *LRRK2* p.G2019S and *GBA1* p.N409S carriers. Our study had three main goals: (1) Define PD penetrance of the *LRRK2*/*GBA1* variants and whether PRS impacts PD penetrance; (2) Explore phenotypic differences among *LRRK2*/*GBA1* carriers; and (3) Evaluate the role of the *APOE* E4 allele in the development of dementia/hallucinations.

## 1 Methods

### 1.1 Participants

Genotyped participants with and without PD were pooled from two online prospective cohorts: (1) the 23andMe Research Database and (2) The Michael J. Fox Foundation's Fox Insight Genetic Substudy (FIGS) [25]. Study recruitment occurred by voluntary participation, website advertisement, and targeted email. 23andMe research participants who self‐reported a diagnosis of PD were invited to participate in Fox Insight, and subsequently FIGS if eligible, between 2017 and 2021 [25]. Fox Insight participants that reported a PD diagnosis at baseline and completed at least one routine longitudinal assessment were invited to enroll in FIGS and were sent 23andMe genotyping kits upon enrollment; however, a small sample (*n* = 27) of FIGS participants without PD were sent a genotyping kit due to unintentional user error or a revision of their diagnosis, and were considered non‐manifest herein. 23andMe research participants that enrolled in FIGS were not re‐genotyped. The data analyzed comprised June and July 2023 data cuts for 23andMe and FIGS data (https://doi.org/10.25549/bxya‐6133), respectively. Both protocols were IRB approved. All participants were US‐based, between 18 and 100 years old, and gave informed consent to participate. See the Data S1 for details.

### 1.2 Phenotypic Data

23andMe participants who self‐reported a diagnosis of PD filled a 196‐item survey designed to capture past and current symptoms. Additional cross‐sectional and longitudinal data were available from 23andMe research participants with and without PD across health/aging update surveys, as well as more disease‐specific and lifestyle surveys. Surveys within FIGS were administered upon enrollment and at predefined intervals [25, 26, 27]. See Table S1 for a detailed list of all aggregated 23andMe and FIGS surveys and their longitudinal cadence.

Age at PD onset was defined as the minimum reported age of PD diagnosis. We excluded participants that reported an age of PD diagnosis < 40 years to create a homogenous cohort [28] and removed outliers with reported ages of PD diagnoses in their teens and 20s (*n* = 1294 total removed, 4%). Furthermore, we excluded participants from modeling if the range of PD diagnosis age varied by > 6 years across surveys. Phenotypic data were summarized by the proportion of participants that ever reported a risk factor or symptom across all timepoints and between both databases. Longitudinal data across both databases were not used to model longitudinal change in symptoms per se, but rather cross‐sectional and longitudinal data (if available) were aggregated within each participant to better estimate whether each participant ever reported a symptom/outcome. For example, a participant who completed a 23andMe survey and responded “no” to a diagnosis of RBD in 2018, but then responded “yes” in a Fox Insight survey in 2020 was considered as reporting a diagnosis of RBD. Table S2 lists all measures used across the 23andMe and FIGS datasets (see Data S1 for details). A diagnosis of mild cognitive impairment (MCI) and reduced arm swing were the only measures that were available in 23andMe surveys and not FIGS.

### 1.3 Risk Factors and Exposures

Risk factors for PD examined included low lifetime caffeine consumption, nonsmoking, family history of PD, prior traumatic brain injury (TBI), and occupational pesticide exposure. Sex (zero = female, one = male) and ancestry were derived from genotyping data [29, 30]. Level of education was binarized into less than Associate degree (zero) and greater than or equal to Associate degree (one).

### 1.4 Genetic Data

DNA extraction and genotyping were performed on saliva samples (see Data S1). *LRRK2* p.G2019S and *GBA1* p.N409S carrier status was established by the presence of pathogenic alleles determined via genotyping. Two SNPs (rs429358 and rs7412) were used to determine the *APOE* haplotype and E4 dosage. A polygenic risk score (PRS) was calculated for each participant using the allelic weights from Nalls et al. [11] PRS including 1805 variants. We excluded variants present in the *LRRK2* (±10 Mb window) and *GBA1* (±1 Mb) loci from the PRS calculation. Principal components (PCs) of genetic ancestry for all participants and European‐specific participants were computed from genotyping data (see Data S1).

### 1.5 Data Analysis

Participants < 40 years of age were excluded from analyses; however, this did not disproportionately impact *GBA1* p.N409S carriers, *χ* ^2^(2) = 1.12, *p* = 0.57. PD cases were stratified according to carrier status (i.e., *LRRK2* p.G2019S, *GBA1* p.N409S, dual carriers, and noncarriers). Dual carriers carried at least one copy of both *LRRK2* p.G2019S and *GBA1* p.N409S. Unless otherwise stated, dual carriers were excluded due to low sample sizes and *n* < five were marked for k‐anonymity [31]. See Table S3 for information on the statistical models, covariates, and sample sizes.

Analyses were performed after excluding related individuals. We computed identical by descent segments of genotyped DNA and excluded second‐degree relatives as recommended by Fahed et al. [32] (see Data S1). Because commercial‐based genetic testing databases generally contain related individuals, and it could be challenging and computationally expensive to identify them, we present the same analyses on the full dataset, without relative exclusion, and report any differences.

#### 1.5.1 Penetrance Estimation

Age of PD diagnosis served as our time‐to‐event (i.e., PD‐free survival) and was the minimum age reported longitudinally (if available). Participants without a PD diagnosis were right censored at the age of their most recent survey completion. To define PD penetrance incident to genetic exposure, we analyzed participants with preexisting and incident diagnoses of PD [15, 33, 34]. We excluded FIGS participants from survival analyses because non‐PD FIGS participants were not genotyped and could upwardly bias penetrance estimations.

We used the Kaplan–Meier method to estimate PD‐free survival probability as a function of carrier status across four levels: (1) *LRRK2* p.G2019S, (2) *GBA1* p.N409S, (3) dual carriers, and (4) noncarriers. Because the effect of carrier status violated the proportional hazard assumption of Cox regression based on Schoenfeld residuals and time, *χ* ^2^(3) = 36.77, *p* = 5.15 × 10^−08^, we used Weibull accelerated failure time (AFT) models to explore the effect of PRS and carrier status on PD‐free survival probability. We performed AFT modeling with and without the covariates of sex and the first 10 ancestry PCs. We repeated the AFT models excluding non‐Europeans using sex and the first five European ancestry PCs as covariates. Predicted survival probabilities and cumulative incidence were generated for males and females with sample means for PRS and ancestry PCs. Male sex was arbitrarily chosen for figures of predicted estimates; however, we also report female cumulative incidence of PD in the corresponding Supporting Information Table.

#### 1.5.2 PD Risk

The risk of PD was examined using two logistic regression analyses [15, 32]. First, we modeled the relative odds of reporting PD as the interaction of carrier status, which included dual carriers, and PRS group: low PRS (1%–25%), middle PRS (25%–75%), and high PRS (75%–100%). Noncarriers with middle PRS served as the reference group. Second, we modeled the relative odds of reporting PD as the interaction of carrier status and PRS decile; noncarriers with median PRS (fifth decile) served as the reference group. Normalized age (*M* = 0, SD = 1), sex, and the first 10 ancestry PCs were used as covariates. Additional sensitivity analyses were performed excluding non‐Europeans (in which case the first five European ancestry specific PCs were used as covariates). We estimated deviations from additivity using ANOVA. Predicted odds ratios were generated using sample means for age and ancestry PCs. As noted above, non‐PD FIGS participants were excluded from PD risk analyses to mitigate the upward bias of risk estimates.

#### 1.5.3 Symptomatic Burden

Logistic regression models estimated the relative odds of reporting each symptom as a function of carrier status. Sufficient sample sizes were unavailable for most symptoms in dual carriers; therefore, the dual carrier models are presented in the Data S1. Noncarriers served as the reference group; covariates included sex, education, PD duration, and age calculated from the date of the latest survey response for each symptom. Using previously described methods [15], we grouped symptoms according to suspected regions of neurodegeneration across the brain: substantia nigra (motor: bradykinesia, shuffling gait, freezing of gait, imbalance, difficulties dressing, reduced arm swing, smaller handwriting, softer speech, tremor), brain stem excluding regions of the pons (autonomic: constipation, erectile dysfunction, nocturia, orthostatic hypotension, increased urinary urgency/frequency), cerebral cortex and limbic areas (cognitive/memory/psychotic: concentration difficulties, MCI diagnosis, difficulties with memory for dates, difficulties with memory in general, hallucinations), olfactory bulb (hyposmia), and areas of the pons for RBD. We adjusted the *p*‐values using false discovery rate within these groupings and used post hoc Tukey‐corrected pairwise comparisons to examine differences between carrier status. We shaded in approximated neuroanatomical regions according to average reported symptom burden within the aforementioned domains. We excluded symptoms from neuroanatomical shading if they contained too few participants (reduced arm swing) or were nonstandard/diffuse (difficulties dressing, general memory difficulties, anxiety, depression). Anxiety and depression were grouped into a psychiatric domain for false discovery rate correction.

#### 1.5.4 APOE Status and Cognitive/Memory/Psychotic Symptoms

The impact of *APOE* haplotype and E4 dosage was assessed across four cognitive/memory/psychotic symptoms: concentration, hallucinations, general memory, and memory for dates. Logistic regressions were used to model the relative odds of each symptom as a function of carrier status and *APOE* haplotype (E3/E3 as reference group). Logistic regression was used to model the relative odds of each symptom as a function of *APOE* E4 dosage (zero, one, two) as a continuous variable, carrier status (noncarriers as reference group), and their interaction. Covariates included sex, education, and PD duration and age calculated from the date of the latest survey response for each symptom.

### 1.6 Software

All analyses were performed using R (v. 4.1.2) [35]. Survival analyses were performed using R packages *survival* [36] and *flexsurv* [37]. Figures were prepared using R packages *ggplot2* [38] and *patchwork* [39].

## 2 Results

### 2.1 PD Penetrance Was Greatest in Dual Carriers

**FIGURE 1:**
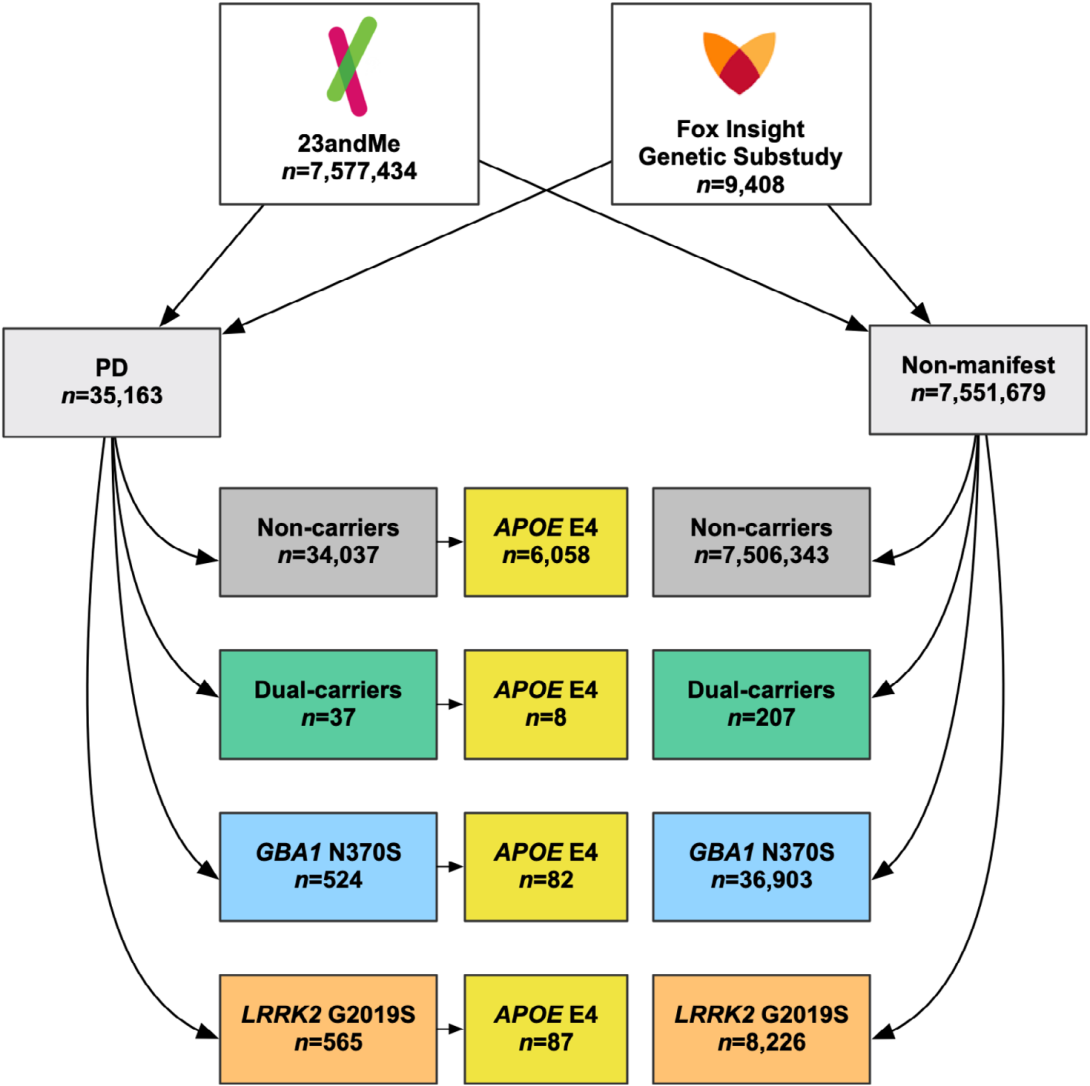
Participant flow diagram. Participants from the 23andMe Research Cohort and the Fox Insight Genetic Substudy (July 2023 data cut) with a clearly defined Parkinson's disease (PD) status and available genotyping data were combined into an analysis of PD symptoms, PD‐free survival, PD risk, and PRS. Sample sizes in the colored boxes represent total possible sample sizes used for the analyses presented. *LRRK2* p.G2019S, *GBA1* p.N409S, dual carriers, and *APOE* E4 carrier groups were defined as carrying at least one pathogenic variant.

The cohort demographics and symptoms are summarized in Table 1 (see Table S4 for total sample sizes), and the study flow in Figure 1. The mean age of PD diagnosis for dual carriers was 60.1 years (SEM = 1.6 years), 61.4 (SEM = 0.5) for *LRRK2* p.G2019S, 59.1 (SEM = 0.5) for *GBA1* p.N409S, and 61.5 (SEM = 0.1) for noncarriers. The Kaplan–Meier analysis, which included *n* = 22,935 PD cases and *n* = 7,343,093 non‐PD controls, is shown in Figure S1. PD‐free survival was different between carrier groups (log‐rank test *χ* ^2^(3) = 3883.00, *p* < 0.001). The cumulative incidence of PD at age 80 was highest in dual carriers (30%), followed by *LRRK2* p.G2019S (24%), *GBA1* p.N409S (4%), and noncarriers (2%; see Table S5). The Weibull AFT models without covariates showed similar results (see Table S6; Figure S2). We observed similar results when excluding related individuals.

**TABLE 1:** Demographics and self‐reported symptoms and risk factors in participants ≥ 40 years of age.

Domain	Measure	Parkinson's disease				Non‐manifest			
		Non carriers	*GBA1* p.N409S	*LRRK2* p.G2019S	Dual carriers	Non‐ carriers	GBA1 p.N409S	*LRRK2* p.G2019S	Dual carriers
Demographics	Male	17,859 (60%)	288 (62%)	261 (51%)	19 (53%)	1,718,411 (42%)	9881 (45%)	2258 (47%)	63 (49%)
	≥ Associate degree	18,395 (71%)	310 (79%)	354 (80%)	25 (76%)	2,515,729 (65%)	15,339 (74%)	3365 (76%)	104 (87%)
	European ancestry	26,597 (89%)	441 (94%)	464 (90%)	36 (100%)	3,294,796 (80%)	19,708 (89%)	3643 (76%)	124 (96%)
	AJ ancestry	1497 (5%)	220 (47%)	299 (58%)	*n* < 5	104,611 (3%)	6696 (30%)	1831 (38%)	99 (77%)
	PD‐free survival age^a^ (years)	62.56 (0.059)	59.85 (0.436)	61.92 (0.424)	60.77 (1.5)	58.22 (0.006)	59.54 (0.079)	59.28 (0.167)	61.59 (1.102)
	PD duration (years)	7.76 (0.033)	8.4 (0.261)	9.73 (0.289)	9.45 (1.034)				
	Age at latest survey (years)	69.97 (0.059)	67.92 (0.446)	71.61 (0.406)	70.41 (1.483)				
Motor	Bradykinesia	5405 (77%)	86 (76%)	181 (77%)	*n* < 5	47,829 (10%)	257 (10%)	199 (12%)	9 (20%)
	Shuffling gait	4390 (62%)	63 (56%)	141 (60%)	*n* < 5	11,274 (2%)	59 (2%)	53 (3%)	*n* < 5
	Freezing of gait	3377 (47%)	55 (49%)	104 (44%)	9 (50%)	5420 (1%)	32 (1%)	29 (2%)	*n* < 5
	Imbalance	5020 (71%)	72 (64%)	165 (70%)	*n* < 5	96,689 (21%)	515 (21%)	333 (20%)	10 (24%)
	Difficulties dressing	3560 (23%)	58 (25%)	58 (22%)	7 (29%)	11 (15%)	*n* < 5	*n* < 5	*n* < 5
	Reduced arm swing	1662 (81%)	*n* < 5	136 (76%)	*n* < 5	25,781 (7%)	100 (5%)	81 (6%)	*n* < 5
	Smaller handwriting	5555 (78%)	91 (80%)	142 (60%)	14 (74%)	10,111 (2%)	57 (2%)	50 (3%)	*n* < 5
	Softer speech	5225 (74%)	79 (72%)	133 (57%)	*n* < 5	18,509 (4%)	109 (4%)	71 (4%)	*n* < 5
	Tremor	5745 (80%)	92 (81%)	195 (82%)	*n* < 5	48,293 (10%)	269 (11%)	192 (11%)	7 (15%)
Non‐motor	Constipation	10,114 (51%)	167 (56%)	183 (49%)	16 (53%)	44,060 (5%)	220 (5%)	81 (6%)	*n* < 5
	Depression	10,453 (37%)	163 (38%)	174 (37%)	10 (28%)	1,068,728 (26%)	5495 (25%)	1211 (26%)	22 (17%)
	Erectile dysfunction	3644 (73%)	54 (69%)	80 (66%)	*n* < 5	7932 (28%)	35 (22%)	88 (27%)	*n* < 5
	Anxiety	5349 (22%)	100 (27%)	101 (25%)	7 (23%)	350,885 (9%)	1962 (9%)	416 (9%)	11 (9%)
	Nocturia	7967 (34%)	133 (38%)	196 (49%)	17 (57%)	30,752 (1%)	176 (1%)	247 (6%)	6 (5%)
	OH	9452 (42%)	153 (44%)	161 (37%)	18 (56%)	37,362 (7%)	189 (6%)	209 (16%)	8 (22%)
	Hyposmia	9075 (54%)	149 (61%)	158 (41%)	13 (45%)	93,161 (6%)	538 (6%)	158 (7%)	*n* < 5
	RBD	6684 (26%)	132 (34%)	62 (14%)	7 (21%)	54,807 (1%)	262 (1%)	69 (2%)	*n* < 5
	Urinary urgency/Freq.	11,434 (60%)	170 (59%)	215 (60%)	18 (62%)	16,538 (21%)	90 (21%)	181 (23%)	5 (21%)
Risk factors	Nonuse of caffeine	1545 (11%)	27 (13%)	34 (14%)	*n* < 5	59,958 (8%)	325 (8%)	75 (9%)	*n* < 5
	Nonuse of coffee	3990 (28%)	54 (25%)	86 (30%)	11 (50%)	215,178 (26%)	1078 (26%)	298 (22%)	11 (24%)
	1° relative with PD	2435 (18%)	35 (18%)	121 (40%)	10 (37%)	31,758 (8%)	244 (12%)	423 (28%)	8 (19%)
	Occupational pesticides	2372 (17%)	26 (12%)	25 (8%)	*n* < 5	28,822 (6%)	126 (5%)	61 (4%)	*n* < 5
	Nonsmoking	15,479 (60%)	243 (63%)	244 (57%)	25 (81%)	2,269,122 (57%)	12,173 (57%)	2604 (57%)	72 (57%)
	TBI	6206 (24%)	64 (16%)	107 (24%)	7 (21%)	196,361 (5%)	951 (4%)	485 (10%)	17 (13%)
Cognitive/Memory	Concentration difficulties	7806 (59%)	135 (70%)	151 (53%)	13 (52%)	131,439 (15%)	651 (14%)	291 (20%)	*n* < 5
	Hallucinations	3423 (26%)	61 (32%)	37 (15%)	*n* < 5	22,465 (3%)	137 (3%)	22 (2%)	*n* < 5
	MCI	1525 (11%)	34 (17%)	14 (5%)	*n* < 5	7644 (2%)	41 (2%)	30 (2%)	*n* < 5
	Memory difficulties: date	1853 (20%)	38 (26%)	51 (20%)	*n* < 5	15,230 (17%)	83 (17%)	158 (19%)	*n* < 5
	Memory difficulties: global	8352 (63%)	141 (73%)	178 (63%)	16 (67%)	194,301 (22%)	1011 (22%)	480 (34%)	10 (24%)

*Note:* Counts are reported as No. (%) for binary variables; continuous measures are reported *M* (SEM). To protect participant privacy, summary statistics, and sample sizes are not reported for *n* < 5.

Abbreviations: AJ, Ashkenazi Jewish; MCI, mild cognitive impairment; OH, orthostatic hypotension; PD, Parkinson's disease; RBD, REM, sleep behavior disorder; TBI, traumatic brain injury.

^a^ Represents the age at the latest survey completion for non‐manifest participants.

### 2.2 High PRS Increases Risk of PD

**FIGURE 2:**
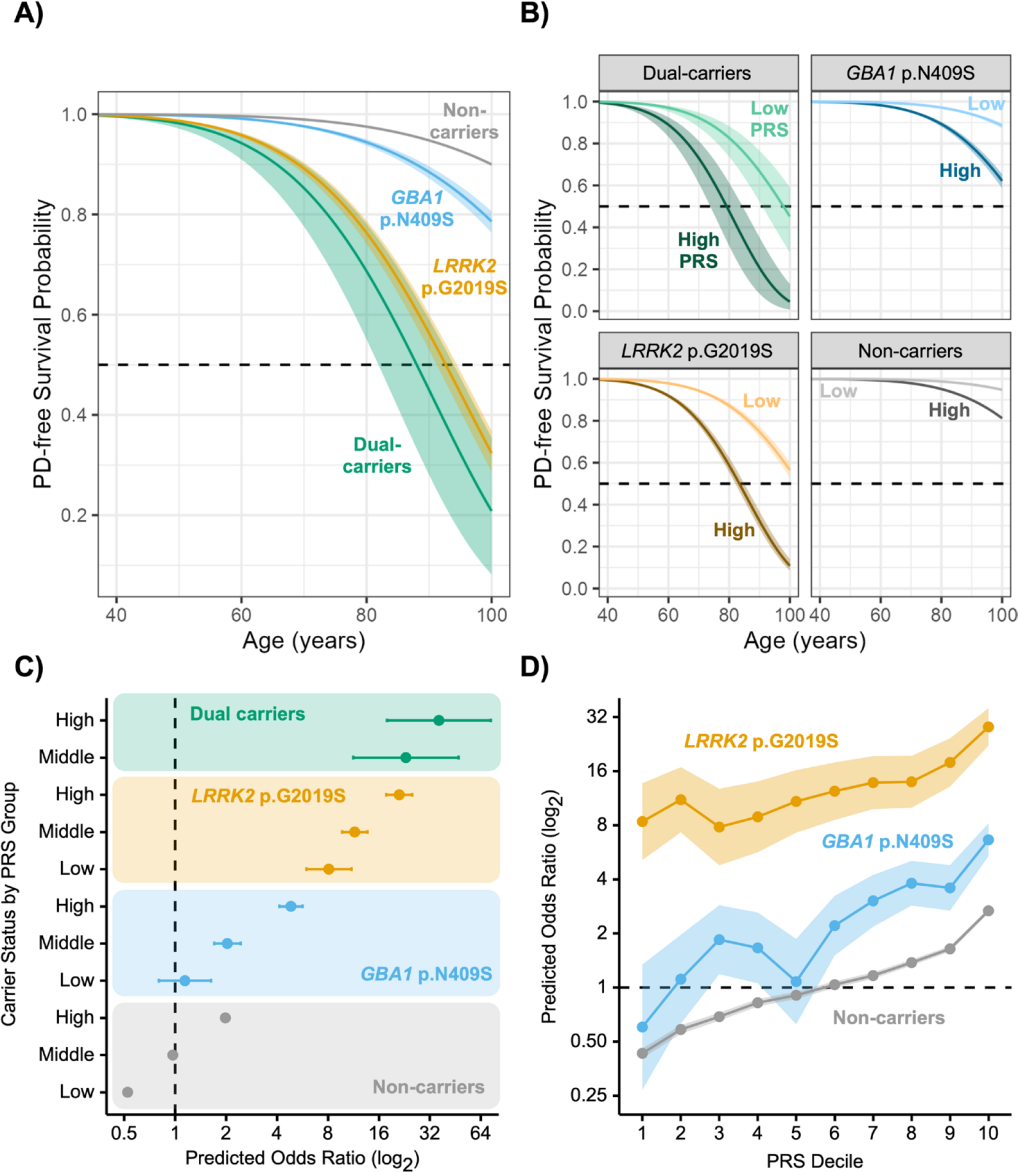
Dual *LRRK2* p.G2019S/*GBA1* p.N409S carriers have the greatest Parkinson's disease (PD) penetrance and PD risk. (A) Curves represent predicted PD‐free survival probability for males using the sample means for polygenic risk score (PRS) and ancestry principal components (PCs). (B) Curves represent predicted PD‐free survival probability for low (10%) and high (90%) PRS, males, and mean PCs. Shading depicts 95% CI. (C) Forest plot of predicted odds ratios indicating a positive association between PD risk and PRS within carrier groups. PRS quantiles defined groups: Low (bottom 25%), middle (between 25%–75%), and high (upper 25%). Dual carriers with low PRS were removed from the plot due to small sample size. Noncarriers with middle PRS were the reference group. Predicted odds ratios were computed for males using sample means of ancestry PCs and age. (D) The predicted relative odds of reporting PD according to PRS decile. Predicted odds ratios were computed as described in (C), but noncarriers at the fifth decile were the reference group. All error bars and shading reflect 95% CIs.

There was a significant effect of PRS on PD‐free survival probability (Figure 2). A greater PRS accelerated the time to PD diagnosis across all carrier groups (TR = 0.921 [0.919, 0.923], *p* < 0.001; see Table S6). An increase in one SD of PRS predicted an earlier age at 50% survival probability by 6.99 [−1.29, 15.26] years for dual carriers, 7.36 [5.18, 9.53] years for *LRRK2* p.G2019S, 9.36 [6.51, 12.21] years for *GBA1* p.N409S, and 10.65 [9.59, 11.71] years for noncarriers (in males with average ancestry PCs). Relative to noncarriers with middle PRS, having a high PRS increased the odds of PD 37‐fold for dual carriers (OR = 37.38 [18.46, 75.66]), 22‐fold for *LRRK2* p.G2019S carriers (OR = 21.78 [18.24, 26.01]), fivefold for *GBA1* p.N409S carriers (OR = 4.99 [4.26, 5.84]), and doubled for noncarriers (OR = 2.04 [1.99, 2.10]; Figure 2C; Table S7). The odds of PD were highest for *LRRK2* p.G2019S and dual carriers regardless of PRS. Relative to the reference group, the risk of PD in noncarriers with high PRS was greater than *GBA1* p.N409S carriers with low PRS. A similar impact of high PRS on disease risk was observed in the decile analysis (Figure 2D; Table S8). Departures from additivity were not observed in the PRS group (low, middle, high) model (ΔDeviance(6) = 9.49, *p* = 0.15 via *χ* ^2^ test) nor in the decile model (ΔDeviance(18) = 22.64, *p* = 0.20). We observed similar results when excluding those with non‐European ancestry (see Tables S8–S12; Figures S2–S4) and the pattern of results was robust to relatedness.

### 2.3 In PD Participants, Greater Prevalence of Non‐Motor Symptoms, Cognitive Impairment, and Hallucinations in *GBA1* p.N409S Relative to *LRRK2* p.G2019S Carriers

**FIGURE 3:**
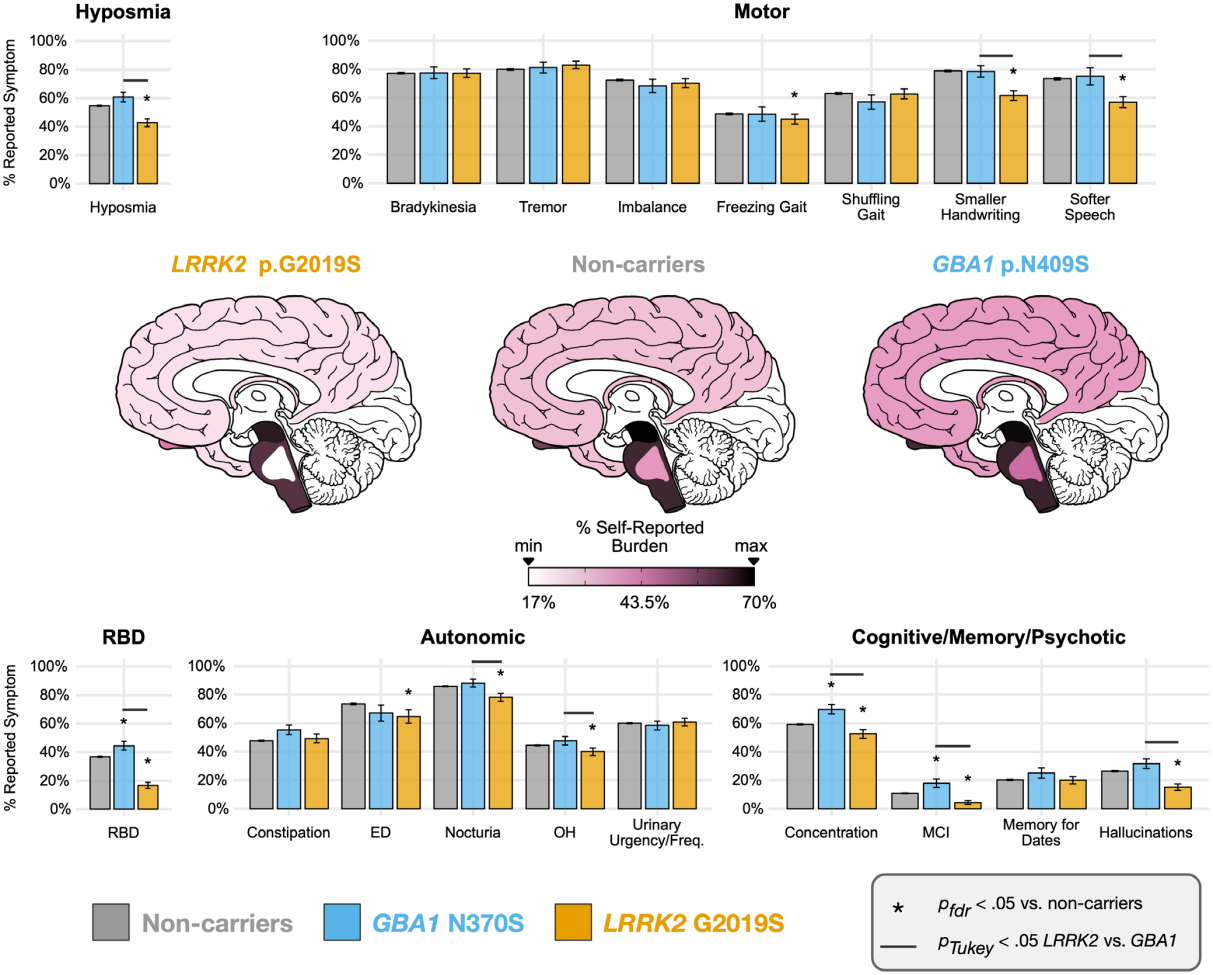
Self‐reported symptom prevalence in *LRRK2* p.G2019S carriers with PD, *GBA1* p.N409S carriers with PD, and noncarriers with PD (idiopathic PD). Symptoms across questionnaires were aggregated into six domains and brain regions were shaded in approximated neuroanatomical regions according to average reported symptom burden across symptoms within domain: Motor (substantia nigra), autonomic (brain stem excluding regions of the pons), cognitive/memory/psychotic (cerebral cortex and limbic areas), hyposmia (olfactory bulb), REM sleep behavior disorder (RBD, areas of the pons). The false discovery rate (FDR) was adjusted in carrier group comparisons to noncarriers within symptom domains. Comparisons between *LRRK2* p.G2019S and *GBA1* p.N409S carriers were adjusted with Tukey's honestly significant difference tests. Dual carrier results are presented in Figure S5. Error bars are SE. Descriptive statistics are not reported for measures with *n* < five due to 23andMe data privacy policies. ED = erectile dysfunction; OH = orthostatic hypotension; MCI = mild cognitive impairment.

The prevalence of symptoms in participants with PD within each neuroanatomical domain for *LRRK2* p.G2019S, *GBA1* p.N409S, and noncarriers is shown in Figure 3 (dual carrier results reported in Figure S5). Odds ratios are presented in Table S13 and post hoc pairwise Tukey tests in Table S14. The prevalence of PD motor symptoms was similar across all carrier groups, with the exception that fewer *LRRK2* p.G2019S PD participants reported hypophonia (softer speech) and micrographia (smaller handwriting) than *GBA1* p.N409S PD participants and noncarriers with PD, and fewer *LRRK2* p.G2019S carriers reported freezing gait relative to noncarriers with PD.

*LRRK2* p.G2019S PD reported less nocturia and orthostatic hypotension than *GBA1* p.N409S PD and noncarriers with PD, and less erectile dysfunction and depression relative to noncarriers with PD. Compared to idiopathic (non‐carrier) PD, the prevalence of RBD was higher in *GBA1* p.N409S PD and lower in *LRRK2* p.G2019S PD. *LRRK2* p.G2019S carriers with PD reported less hyposmia relative to both *GBA1* p.N409S PD and noncarriers with PD. Relative to noncarriers with PD, *GBA1* p.N409S PD reported more concentration and problems with their memory in general, and were more likely to be diagnosed with MCI. *LRRK2* p.G2019S PD reported fewer issues with concentration, fewer hallucinations, and fewer MCI diagnoses. Cognitive symptoms were not more severe in dual carriers (see Tables S15 and S16; Figure S5). When excluding for relatedness, we observed different results for a few symptoms. *GBA1* p.N409S carriers were more likely to report hallucinations compared to noncarriers, and reported RBD at similar prevalence to noncarriers. *LRRK2* p.G2019S carriers were more likely to report urinary frequency/urgency symptoms compared to noncarriers, and reported diagnoses of depression at similar prevalence to noncarriers (see Data S1).

### 2.4 *APOE* E4 Dosage Increases Risk of Cognitive and Psychotic Symptoms in *LRRK2* p.G2019S and *GBA1* p.N409S Carriers With PD

**FIGURE 4:**
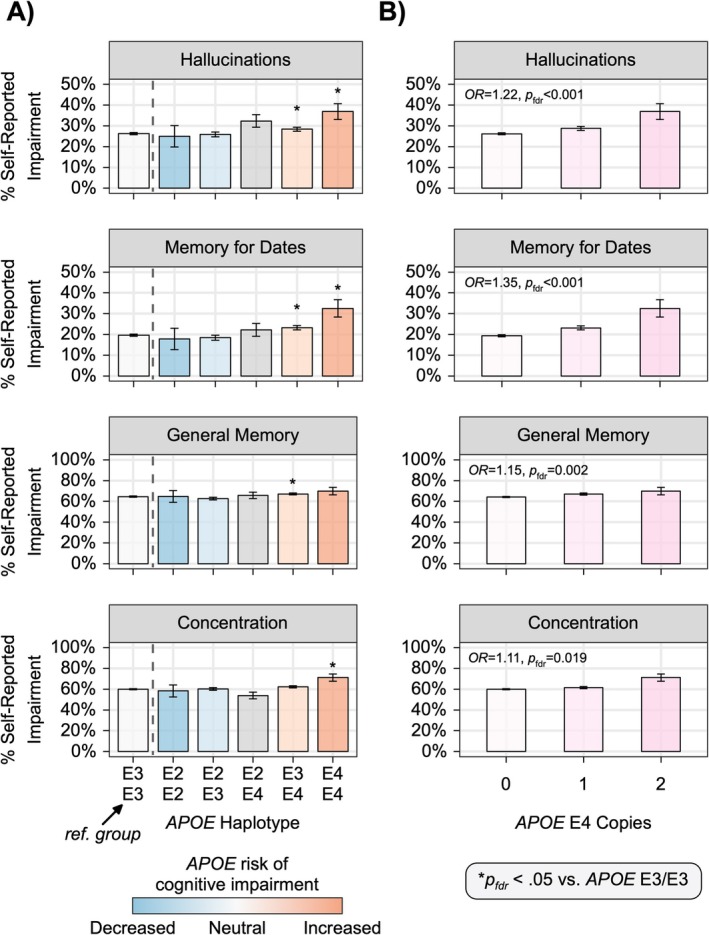
The effect of APOE haplotype and E4 dosage on cognitive/memory/psychotic symptoms in individuals with Parkinson's disease (PD). (A) The percentage of participants with PD with cognitive/memory/psychotic symptoms across possible *APOE* haplotypes collapsed across carrier status. Stars indicate significantly greater relative odds for reporting symptoms compared to *APOE* E3/E3 individuals (i.e., reference group). Bars are color coded by hypothesized risk of cognitive impairment due to *APOE* haplotype; E2/E4 carriers carry one protective (E2) and one risk variant (E4) and are therefore colored gray. (B) Bar graphs show a positive association between *APOE* E4 dosage and percentage of cognitive/memory/psychotic symptoms in participants with PD collapsed across carrier status. Error bars are SE.

The presence of the *APOE* E4 allele status (haplotype) was associated with cognitive, memory, and psychotic symptoms (Table S17; Figure 4A). Each additional copy of the *APOE* E4 allele conferred a 22% increase in the odds of reporting hallucinations (Table S18; Figure 4B). Similarly, *APOE* E4 dosage was associated with greater odds of reporting memory and concentration issues after adjusting for age, sex, education, and PD duration. We did not observe any interactions between *APOE* E4 dosage and carrier status, suggesting that *APOE* E4 dosage contributes an additive risk (on the log odds scale) to cognitive/memory/psychotic symptoms in *LRRK2* p.G2019S and *GBA1* p.N409S carriers with PD; however, we were underpowered to fully test this hypothesis (see Table S19 for sample sizes). All results were robust to relatedness except for the effect of *APOE* E4 on reported difficulties with concentration (see Data S1).

## 3 Discussion

This large‐cohort analysis demonstrates that *LRRK2* p.G2019S is six times more penetrant than the *GBA1* p.N409S variant. The penetrance of PD was highest for dual carriers and *LRRK2* p.G2019S carriers. PRS modifies PD risk, such that *GBA1* p.N409S carriers with low PRS have similar PD risk as noncarriers with median PRS. Carriage of *APOE* E4 variants increases the risk of cognitive impairment in PD, whereas the *LRRK2* p.G2019S variant reduces the risk of hallucinations and dementia.

We show that by the age of 80 years, 30% of dual carriers, 24% of LRRK2 p. G2019S carriers, and 4% of *GBA1* p.N409S carriers developed PD, compared to 2% of noncarriers. Our observed cumulative incidence of PD for carriers is less than previous reports. The penetrance of PD ranges between 11% and 30% for *GBA1* carriers [40, 41] and between 32% and 80% for *LRRK2* p.G2019S carriers [42, 43, 44]. We previously observed a 49% cumulative incidence of PD in *LRRK2* p.G2019S carriers by the age of 80 [15]. This lower PD incidence in the current investigation likely resulted from using a large non‐manifest cohort and differences in recruitment. In contrast to our previous work that selectively recruited *LRRK2* p.G2019S carriers [15], the current study had different entry criteria. The 23andMe and FIGS cohorts are nonclinical, volunteer, self‐report samples that differ from clinic‐based cohorts that may overestimate penetrance [44]. Statistical methodology had little to no effect on penetrance estimation, as estimates were similar between Kaplan–Meier and AFT modeling.

Although dual carriers have a high cumulative incidence of PD, the combination of both variants is extremely rare and accounted for only 1:950 PD cases. *LRRK2* p.G2019S, although highly penetrant, had a carrier rate of 1:58 PD cases, whereas *GBA1* p.N409S was less penetrant but had a more frequent carrier rate of 1:63 PD cases. Of those participants that reported a family history of PD (i.e., first degree relative), the frequency of *LRRK2* p.G2019S carriers was 1.66% (SE = 0.07%), 0.08% (SE = 0.05%) for *GBA1* p.N409S, 0.05% (SE = 0.01%) for dual carriers, and 97.50% (SE = 0.08%) for noncarriers. The frequency of reporting no family history of PD decreased within carrier groups: 0.38% (SE = 0.01%) *LRRK2* p.G2019S carriers, 0.51% (SE = 0.01%) *GBA1* p.N409S, 0.01% (SE = 0.002%) dual carriers, and 99.09% (SE = 0.01%) for noncarriers. Although *GBA1* variants are a common genetic risk factor for PD [6], additional risk markers of imminent phenoconversion are needed to enrich early interventional clinical trials. *GBA1* variants have also been linked to cases of DLB and multiple system atrophy [45], which underlines the importance of finding biomarkers to optimize the distinction between the different α‐synucleinopathies in the early stages.

Our findings show the risk of cognitive impairment is highest in *GBA1* p.N409S carriers. Based on the anatomical models, we show that while *GBA1* p.N409S is considered a “mild” variant, it is associated with widespread neuronal loss beyond the substantia nigra. This is consistent with clinical cohorts [46, 47, 48, 49, 50, 51, 52] and pathology studies showing Lewy body pathology throughout the cortex in *GBA1* carriers [23, 53, 54, 55]. A novel finding in our study was that each additional copy of *APOE* E4 conferred between 11% and 35% increase in the odds of reporting hallucinations and cognitive impairment in PD. This adds to the risk of a more severe cognitive phenotype in *GBA1* p.N409S carriers, and may be useful when tracking progression [23, 56]. In contrast to others [56], we did not find evidence suggesting that the *APOE* E2 allele was protective.

Our data add to the growing evidence that *LRRK2* appears to reduce the risk of non‐motor symptoms. First, we show that *LRRK2* p.G2019S PD has the lowest prevalence of hallucinations, RBD, hyposmia, nocturia, orthostatic hypotension, difficulties with concentration, and MCI diagnoses. Second, we show that dual carriers do not report more severe symptoms than *GBA1* carriers across all non‐motor symptom domains (see Figure S5). This aligns with other reports that suggest the presence of the gain of function *LRRK2* p.G2019S variant offsets the down regulation of GCase [57, 58, 59]. While α‐synuclein Lewy body cortical involvement may not always predict dementia [60], it is worthwhile noting that at least one‐third of *LRRK2* carriers do not have evidence of α‐synuclein seeding in the CSF [9] and do not show CNS Lewy bodies at autopsy [61, 62] which suggests a restricted neurodegenerative process that is independent of α‐synuclein and slower progressing [15, 63, 64, 65, 66].

A major obstacle for early interventional disease‐modifying clinical trials is the lack of enrichment [67]. One promising enrichment strategy is PRS. PRS can heighten the risk of phenoconversion at an early age [11, 13, 68, 69], including in carriers of genetic risk variants [14, 15, 70], and aid in balancing treatment arms [71]. As shown by our data, PRS affects PD penetrance more than sex, low caffeine intake, nonsmoking, and TBI [15]. We observed that noncarriers at the top decile for PRS had nearly a threefold increase in the relative odds of reporting PD compared to noncarriers with median PRS. In contrast, *GBA1* p.N409S carriers at the top decile for PRS experienced a sevenfold increase in the relative odds of reporting PD compared to noncarriers with median PRS, which may help reduce the number of participants required for *GBA1* trials. An even greater risk was seen in *LRRK2* p.G2019S carriers, who experienced a 31‐fold increase at the top PRS decile relative to noncarriers with median PRS. Our results align with previous studies, showing that a greater PRS accelerates the age of diagnosis, resulting in a greater incidence of PD [13] and resulting in greater risk of PD within carrier groups [14, 72]. However, given the rarity of the *LRRK2* p.G2019S variant, too strict a PRS‐based enrichment for *LRRK2* trials would make it difficult to recruit a sufficient number of candidates in a timely manner.

There are some limitations to our study. The 23andMe cohort is unique, assembled by virtue of study participants being interested in health‐related genetics, and likely more affluent, which may limit the generalizability of the results, including underrepresented groups or diverse populations. The PD status and symptom prevalence in both the Fox Insight and 23andMe databases is based on self‐report, creating the potential for bias and inaccuracy, especially with regard to the prevalence of symptoms. For example, participants with cognitive impairment may have inaccurately reported their symptoms or not completed the questionnaires; however, 23andMe PD self‐report measures have shown excellent correlation with clinical evaluations [73, 74, 75]. From examining the number of participants with PD that viewed their 23andMe genetic health risk reports, 46% were aware of their *LRRK2* p.G2019S and *GBA1* p.N409S carrier status, and 32% were aware of their *APOE* E4 carrier status. Therefore, it is possible that the participants who were aware of their genetic predisposition experienced recall bias, i.e., reported increased symptom burden or cognitive symptoms. We excluded loci near *LRRK2* and *GBA1* in the PRS calculation to reduce potential confounding factors; however, this may have unintentionally excluded important risk variants that affect PD outcomes.

To create a more homogenized cohort, we restricted the age of PD diagnosis to ≥ 40 years, which eliminated a small minority of early onset cases. Although similar in content, some of the questions used to assess symptoms across the 23andMe and FIGS datasets were not exact matches (e.g., hallucinations, hyposmia) and queried participants across different periods of recall (e.g., In the past week…; In the past month…). These differences could have introduced increased variability in response and operational definitions of symptoms. We were unable to integrate biomarker data (e.g., alpha‐synuclein and tau) that support findings on cognitive symptoms and dementia. Future research would be well served to incorporate biological measures to identify potential biomarkers and identify mechanistic explanations for the differences in etiology between carriers of genetic risk variants with PD. Additionally, it is possible that PD penetrance was underestimated by not directly assessing subtle signs of parkinsonism in non‐PD controls. Future work should also interrogate additional variants, including *GBA1* p.E365K (formerly p.E326K), to compare their relative penetrance and symptom profiles.

## 4 Conclusion

Our findings support the use of genetic screening and PRS to enrich candidate selection for neuroprotective trials. The distinct pathways affected by *LRRK2* and *GBA1* mutations may offer insights into the pathobiology of PD and strategies for treating progression.

## Author Contributions

M.J.K., S.A., L.N.‐K. contributed to the study's conception and design; M.J.K., M.V.H., P.F., J.S., A.G., S.T., S.M., D.A.H., S.A., L.N.‐K., and the 23andMe Research Team contributed to data acquisition and analysis; and M.J.K., M.V.H., P.F., G.M.R., R.B.S., J.S., A.G., S.T., S.M., K.S., J.G., D.A.H., J.Y.T., S.A., and L.N.‐K. contributed to drafting the text and/or preparing the figures.

## Conflicts of Interest

At the time of their contributions, the following authors were employed by and/or held stock or stock options in 23andMe Inc.: M.J.K., M.V.H., P.F., J.S., A.G., S.T., S.M., K.S., D.A.H., J.Y.T., S.A., L.N.K.

## Supporting information


**Data S1:** Supplementary Material.

**Tables S1‐S19:** Supporting Tables.

## Data Availability

Model outputs for all logistic regressions and survival models are provided as Supporting Information Tables. Individual‐level data from 23andMe are not publicly available due to participant confidentiality and in accordance with the IRB‐approved protocol under which the study was conducted. The Fox Insight Genetic Substudy participant data are available through Fox DEN (https://foxden.michaeljfox.org). No custom code or software was generated as part of the study. Details of all software packages used for data processing and analysis may be found in the Methods.

## References

[1] E. Menozzi , A. H. V. Schapira , F. Blandini , and M. Avenali , “Who Is at Risk of Parkinson Disease? Refining the Preclinical Phase of GBA1 and LRRK2 Variant Carriers: A Clinical, Biochemical, and Imaging Approach,” Current Neurology and Neuroscience Reports 23 (2023): 121–130, 10.1007/s11910-023-01259-1. 36881256 PMC10119235

[2] E. Tolosa , M. Vila , C. Klein , and O. Rascol , “LRRK2 in Parkinson Disease: Challenges of Clinical Trials,” Nature Reviews. Neurology 16, no. 2 (2020): 97–107. 31980808 10.1038/s41582-019-0301-2

[3] D. G. Healy , M. Falchi , S. S. O'Sullivan , et al., “Phenotype, Genotype, and Worldwide Genetic Penetrance of LRRK2‐Associated Parkinson's Disease: A Case‐Control Study,” Lancet Neurology 7, no. 7 (2008): 583–590. 18539534 10.1016/S1474-4422(08)70117-0 PMC2832754

[4] E. Hertz , Y. Chen , and E. Sidransky , “Gaucher Disease Provides a Unique Window Into Parkinson Disease Pathogenesis,” Nature Reviews. Neurology 20, no. 9 (2024): 526–540. 39107435 10.1038/s41582-024-00999-z

[5] E. M. Rocha , M. T. Keeney , R. Di Maio , et al., “LRRK2 and Idiopathic Parkinson's Disease,” Trends in Neurosciences 45, no. 3 (2022): 224–236. 34991886 10.1016/j.tins.2021.12.002 PMC8854345

[6] E. Sidransky , M. A. Nalls , J. O. Aasly , et al., “Multicenter Analysis of Glucocerebrosidase Mutations in Parkinson's Disease,” New England Journal of Medicine 361, no. 17 (2009): 1651–1661. 19846850 10.1056/NEJMoa0901281 PMC2856322

[7] J.‐M. Taymans , M. Fell , T. Greenamyre , et al., “Perspective on the Current State of the LRRK2 Field,” npj Parkinson's Disease 9, no. 1 (2023): 1–9. 10.1038/s41531-023-00544-7 PMC10314919 37393318

[8] R. Saunders‐Pullman , A. Mirelman , R. N. Alcalay , et al., “Progression in the LRRK2‐Associated Parkinson Disease Population,” JAMA Neurology 75, no. 3 (2018): 312–319. 29309488 10.1001/jamaneurol.2017.4019 PMC5885854

[9] A. Siderowf , L. Concha‐Marambio , D.‐E. Lafontant , et al., “Assessment of Heterogeneity Among Participants in the Parkinson's Progression Markers Initiative Cohort Using α‐Synuclein Seed Amplification: A Cross‐Sectional Study,” Lancet Neurology 22, no. 5 (2023): 407–417. 37059509 10.1016/S1474-4422(23)00109-6 PMC10627170

[10] J. J. Kim , D. Vitale , D. V. Otani , et al., “Multi‐Ancestry Genome‐Wide Association Meta‐Analysis of Parkinson's Disease,” Nature Genetics 56, no. 1 (2024): 27–36. 38155330 10.1038/s41588-023-01584-8 PMC10786718

[11] M. A. Nalls , C. Blauwendraat , C. L. Vallerga , et al., “Identification of Novel Risk Loci, Causal Insights, and Heritable Risk for Parkinson's Disease: A Meta‐Analysis of Genome‐Wide Association Studies,” Lancet Neurology 18, no. 12 (2019): 1091–1102. 31701892 10.1016/S1474-4422(19)30320-5 PMC8422160

[12] M. Rizig , S. Bandres‐Ciga , M. B. Makarious , et al., “Identification of Genetic Risk Loci and Causal Insights Associated With Parkinson's Disease in African and African Admixed Populations: A Genome‐Wide Association Study,” Lancet Neurology 22, no. 11 (2023): 1015–1025. 37633302 10.1016/S1474-4422(23)00283-1 PMC10593199

[13] O. Goldstein , S. Shani , M. Gana‐Weisz , et al., “The Effect of Polygenic Risk Score on PD Risk and Phenotype in *LRRK2* G2019S and *GBA1* Carriers,” Journal of Parkinson's Disease 15 (2025): 291–299. 10.1177/1877718X241310722 PMC13347430 39973498

[14] H. Iwaki , C. Blauwendraat , M. B. Makarious , et al., “Penetrance of Parkinson's Disease in LRRK2 p.G2019S Carriers Is Modified by a Polygenic Risk Score,” Movement Disorders 35, no. 5 (2020): 774–780. 31958187 10.1002/mds.27974 PMC8975556

[15] M. J. Kmiecik , S. Micheletti , D. Coker , et al., “Genetic Analysis and Natural History of Parkinson's Disease due to the LRRK2 G2019S Variant,” Brain 147, no. 6 (2024): 1996–2008. 38804604 10.1093/brain/awae073 PMC11146432

[16] G. M. Riboldi and A. B. Di Fonzo , “GBA, Gaucher Disease, and Parkinson's Disease: From Genetic to Clinic to New Therapeutic Approaches,” Cells 8, no. 4 (2019): 364. 31010158 10.3390/cells8040364 PMC6523296

[17] L. Y. Wu , R. Real , A. Martinez‐Carrasco , et al., “Investigation of the Genetic Aetiology of Lewy Body Diseases With and Without Dementia,” Brain Communications 6, no. 4 (2024): fcae190. 38978726 10.1093/braincomms/fcae190 PMC11228432

[18] S. Emrani , H. A. Arain , C. DeMarshall , and T. Nuriel , “APOE4 Is Associated With Cognitive and Pathological Heterogeneity in Patients With Alzheimer's Disease: A Systematic Review,” Alzheimer's Research & Therapy 12, no. 1 (2020): 141. 10.1186/s13195-020-00712-4 PMC7643479 33148345

[19] J. Fortea , J. Pegueroles , D. Alcolea , et al., “APOE4 Homozygozity Represents a Distinct Genetic Form of Alzheimer's Disease,” Nature Medicine 30, no. 7 (2024): 2093. 10.1038/s41591-024-02931-w PMC13310155 38710950

[20] J. L. Robinson , E. B. Lee , S. X. Xie , et al., “Neurodegenerative Disease Concomitant Proteinopathies Are Prevalent, Age‐Related and APOE4‐Associated,” Brain 141, no. 7 (2018): 2181–2193. 29878075 10.1093/brain/awy146 PMC6022546

[21] R. Chia , M. S. Sabir , S. Bandres‐Ciga , et al., “Genome Sequencing Analysis Identifies New Loci Associated With Lewy Body Dementia and Provides Insights Into Its Genetic Architecture,” Nature Genetics 53, no. 3 (2021): 294–303. 33589841 10.1038/s41588-021-00785-3 PMC7946812

[22] R. Real , A. Martinez‐Carrasco , R. H. Reynolds , et al., “Association Between the LRP1B and APOE Loci and the Development of Parkinson's Disease Dementia,” Brain 146, no. 5 (2023): 1873–1887. 36348503 10.1093/brain/awac414 PMC10151192

[23] D. J. Irwin , M. T. White , J. B. Toledo , et al., “Neuropathologic Substrates of Parkinson Disease Dementia,” Annals of Neurology 72, no. 4 (2012): 587–598. 23037886 10.1002/ana.23659 PMC3484250

[24] D. Tsuang , J. B. Leverenz , O. L. Lopez , et al., “APOE ϵ4 Increases Risk for Dementia in Pure Synucleinopathies,” JAMA Neurology 70, no. 2 (2013): 223–228. 23407718 10.1001/jamaneurol.2013.600 PMC3580799

[25] J. Gottesman , Y. Karim , J. Forbes , et al., “Fox Insight at 5 Years—A Cohort of 54,000 Participants Contributing Longitudinal Patient‐Reported Outcome, Genetic, and Microbiome Data Relating to Parkinson's Disease,” Scientific Data 11, no. 1 (2024): 615. 38866856 10.1038/s41597-024-03407-9 PMC11169221

[26] M. J. Kmiecik , “Fox Insight Survey Timeline” (2024), Zenodo, https://zenodo.org/records/10999653.

[27] Michael J. Fox Foundation for Parkinson's Research , “Fox Inisght Participant Schedule of Activities” (2023), https://foxden.michaeljfox.org/insight/resources/Participant_Schedule_of_Activities_2023‐12‐08.pdf.

[28] B. Post , L. van den Heuvel , T. van Prooije , X. van Ruissen , B. van de Warrenburg , and J. Nonnekes , “Young Onset Parkinson's Disease: A Modern and Tailored Approach,” Journal of Parkinson's Disease 10, no. s1 (2020): S29–S36. 10.3233/JPD-202135 PMC7592661 32651336

[29] 23andMe, Inc ., “Ancestry Composition: 23andMe's State‐of‐the‐Art Geographic Ancestry Analysis” (2022), https://www.23andme.com/ancestry‐composition‐guide/.

[30] E. Y. Durand , C. B. Do , J. L. Mountain , and J. M. Macpherson , “Ancestry Composition: A Novel, Efficient Pipeline for Ancestry Deconvolution” (2014), 010512, https://www.biorxiv.org/content/10.1101/010512v1.

[31] L. Sweeney , “k‐ANONYMITY: A MODEL FOR PROTECTING PRIVACY,” International Journal of Uncertainty, Fuzziness and Knowledge‐Based Systems 10, no. 5 (2002): 557–570.

[32] A. C. Fahed , M. Wang , J. R. Homburger , et al., “Polygenic Background Modifies Penetrance of Monogenic Variants for Tier 1 Genomic Conditions,” Nature Communications 11, no. 1 (2020): 3635. 10.1038/s41467-020-17374-3 PMC7441381 32820175

[33] D. J. Thompson , D. Wells , S. Selzam , et al., “UK Biobank Release and Systematic Evaluation of Optimised Polygenic Risk Scores for 53 Diseases and Quantitative Traits” (2022), 10.1101/2022.06.16.22276246v2.

[34] W. Wang , N. Eriksson , M. McIntyre , et al., “Prospective Analysis of Incident Disease Among Individuals of Diverse Ancestries Using Genetic and Conventional Risk Factors” (2023), 10.1101/2023.10.23.23297414v1.

[35] R Core Team , R: A Language and Environment for Statistical Computing (R Foundation for Statistical Computing, 2025), https://www.R‐project.org/.

[36] T. Therneau , “A Package for Survival Analysis in R” (2020), https://CRAN.R‐project.org/package=survival.

[37] C. Jackson , “Flexsurv: A Platform for Parametric Survival Modeling in R,” Journal of Statistical Software 70 (2016): 1–33. 10.18637/jss.v070.i08 PMC5868723 29593450

[38] H. Wickham , ggplot2: Elegant Graphics for Data Analysis (Springer‐Verlag, 2016).

[39] T. L. Pederson , “Patchwork: The Composer of Plots” (2019), https://CRAN.R‐project.org/package=patchwork.

[40] M. Anheim , A. Elbaz , S. Lesage , et al., “Penetrance of Parkinson Disease in Glucocerebrosidase Gene Mutation Carriers,” Neurology 78, no. 6 (2012): 417–420. 22282650 10.1212/WNL.0b013e318245f476

[41] H. Q. Rana , M. Balwani , L. Bier , and R. N. Alcalay , “Age‐Specific Parkinson Disease Risk in *GBA* Mutation Carriers: Information for Genetic Counseling,” Genetics in Medicine 15, no. 2 (2013): 146–149. 22935721 10.1038/gim.2012.107 PMC3519952

[42] S. Goldwurm , M. Zini , L. Mariani , et al., “Evaluation of LRRK2 G2019S Penetrance,” Neurology 68, no. 14 (2007): 1141–1143. 17215492 10.1212/01.wnl.0000254483.19854.ef

[43] J. C. Latourelle , M. Sun , M. F. Lew , et al., “The Gly2019Ser Mutation in LRRK2is Not Fully Penetrant in Familial Parkinson's Disease: The GenePD Study,” BMC Medicine 6, no. 1 (2008): 32. 18986508 10.1186/1741-7015-6-32 PMC2596771

[44] J. Trinh , R. Amouri , J. E. Duda , et al., “A Comparative Study of Parkinson's Disease and Leucine‐Rich Repeat Kinase 2 p.G2019S Parkinsonism,” Neurobiology of Aging 35, no. 5 (2014): 1125–1131. 24355527 10.1016/j.neurobiolaging.2013.11.015

[45] S. Koga , F. Li , N. Zhao , et al., “Clinicopathologic and Genetic Features of Multiple System Atrophy With Lewy Body Disease,” Brain Pathology 30, no. 4 (2020): 766–778. 32232888 10.1111/bpa.12839 PMC7383746

[46] R. N. Alcalay , E. Caccappolo , H. Mejia‐Santana , et al., “Cognitive Performance of GBA Mutation Carriers With Early‐Onset PD,” Neurology 78, no. 18 (2012): 1434–1440. 22442429 10.1212/WNL.0b013e318253d54b PMC3345785

[47] K. Brockmann , K. Srulijes , A.‐K. Hauser , et al., “GBA‐Associated PD Presents With Nonmotor Characteristics,” Neurology 77, no. 3 (2011): 276–280. 21734182 10.1212/WNL.0b013e318225ab77

[48] R. Cilia , S. Tunesi , G. Marotta , et al., “Survival and Dementia in ‐Associated Parkinson's Disease: The Mutation Matters,” Annals of Neurology 80, no. 5 (2016): 662–673. 27632223 10.1002/ana.24777

[49] S. Jesús , I. Huertas , I. Bernal‐Bernal , et al., “GBA Variants Influence Motor and Non‐Motor Features of Parkinson's Disease,” PLoS One 11, no. 12 (2016): e0167749. 28030538 10.1371/journal.pone.0167749 PMC5193380

[50] T. Kozlovski , A. Mitelpunkt , A. Thaler , et al., “Hierarchical Data‐Driven Analysis of Clinical Symptoms Among Patients With Parkinson's Disease,” Frontiers in Neurology 10 (2019): 531, 10.3389/fneur.2019.00531/full. 31164863 PMC6536639

[51] J. Ren , G. Zhou , Y. Wang , et al., “Association of GBA Genotype With Motor and Cognitive Decline in Chinese Parkinson's Disease Patients,” Frontiers in Aging Neuroscience 15 (2023): 1091919, 10.3389/fnagi.2023.1091919/full. 36845659 PMC9950580

[52] M. Toffoli , H. Chohan , S. Mullin , et al., “Phenotypic Effect of *GBA1* Variants in Individuals With and Without Parkinson's Disease: The RAPSODI Study,” Neurobiology of Disease 188 (2023): 106343. 37926171 10.1016/j.nbd.2023.106343

[53] A. L. Gündner , G. Duran‐Pacheco , S. Zimmermann , et al., “Path Mediation Analysis Reveals GBA Impacts Lewy Body Disease Status by Increasing α‐Synuclein Levels,” Neurobiology of Disease 121 (2019): 205–213. 30236861 10.1016/j.nbd.2018.09.015

[54] P. A. Kempster , S. S. O'Sullivan , J. L. Holton , et al., “Relationships Between Age and Late Progression of Parkinson's Disease: A Clinico‐Pathological Study,” Brain 133, no. 6 (2010): 1755–1762. 20371510 10.1093/brain/awq059

[55] M. Selikhova , D. R. Williams , P. A. Kempster , J. L. Holton , T. Revesz , and A. J. Lees , “A Clinico‐Pathological Study of Subtypes in Parkinson's Disease,” Brain 132, no. 11 (2009): 2947–2957. 19759203 10.1093/brain/awp234

[56] N. U. Okubadejo , O. Okunoye , O. O. Ojo , et al., “APOE E4 Is Associated With Impaired Self‐Declared Cognition but Not Disease Risk or Age of Onset in Nigerians With Parkinson's Disease,” npj Parkinson's Disease 8, no. 1 (2022): 1–6. 10.1038/s41531-022-00411-x PMC9653490 36371506

[57] N. Omer , N. Giladi , T. Gurevich , et al., “A Possible Modifying Effect of the G2019S Mutation in the LRRK2 Gene on GBA Parkinson's Disease,” Movement Disorders 35, no. 7 (2020): 1249–1253. 32353202 10.1002/mds.28066

[58] R. A. Ortega , C. Wang , D. Raymond , et al., “Association of Dual LRRK2 G2019S and GBA Variations With Parkinson Disease Progression,” JAMA Network Open 4, no. 4 (2021): e215845. 33881531 10.1001/jamanetworkopen.2021.5845 PMC8060834

[59] G. Yahalom , L. Greenbaum , S. Israeli‐Korn , et al., “Carriers of Both GBA and LRRK2 Mutations, Compared to Carriers of Either, in Parkinson's Disease: Risk Estimates and Genotype‐Phenotype Correlations,” Parkinsonism & Related Disorders 62 (2019): 179–184. 30573413 10.1016/j.parkreldis.2018.12.014

[60] C. Colosimo , A. J. Hughes , L. Kilford , and A. J. Lees , “Lewy Body Cortical Involvement May Not Always Predict Dementia in Parkinson's Disease,” Journal of Neurology, Neurosurgery, and Psychiatry 74, no. 7 (2003): 852–856. 12810766 10.1136/jnnp.74.7.852 PMC1738521

[61] C. Gaig , M. J. Martí , M. Ezquerra , et al., “G2019S LRRK2 Mutation Causing Parkinson's Disease Without Lewy Bodies,” Journal of Neurology, Neurosurgery, and Psychiatry 78, no. 6 (2007): 626–628. 17210620 10.1136/jnnp.2006.107904 PMC2077973

[62] L. V. Kalia , A. E. Lang , L.‐N. Hazrati , et al., “Clinical Correlations With Lewy Body Pathology in LRRK2‐Related Parkinson Disease,” JAMA Neurology 72, no. 1 (2015): 100–105. 25401511 10.1001/jamaneurol.2014.2704 PMC4399368

[63] S. Ben Romdhan , N. Farhat , A. Nasri , et al., “LRRK2 G2019S Parkinson's Disease With More Benign Phenotype Than Idiopathic,” Acta Neurologica Scandinavica 138, no. 5 (2018): 425–431. 29989150 10.1111/ane.12996

[64] C. Gaig , D. Vilas , J. Infante , et al., “Nonmotor Symptoms in LRRK2 G2019S Associated Parkinson's Disease,” PLoS One 9, no. 10 (2014): e108982. 25330404 10.1371/journal.pone.0108982 PMC4201457

[65] M. Kestenbaum and R. N. Alcalay , “Clinical Features of LRRK2 Carriers With Parkinson's Disease,” in Leucine‐Rich Repeat Kinase 2 (LRRK2), ed. H. J. Rideout (Springer International Publishing, 2017), 31–48, 10.1007/978-3-319-49969-7_2. 28353277

[66] S. Srivatsal , B. Cholerton , J. B. Leverenz , et al., “Cognitive Profile of LRRK2‐Related Parkinson's Disease,” Movement Disorders 30, no. 5 (2015): 728–733. 25650144 10.1002/mds.26161 PMC4397146

[67] C. H. de Aquino , “Methodological Issues in Randomized Clinical Trials for Prodromal Alzheimer's and Parkinson's Disease,” Frontiers in Neurology 12 (2021): 694329, 10.3389/fneur.2021.694329/full. 34421799 PMC8377160

[68] V. Escott‐Price , Consortium for the IPDG , M. A. Nalls , et al., “Polygenic Risk of Parkinson Disease Is Correlated With Disease Age at Onset,” Annals of Neurology 77, no. 4 (2015): 582–591. 25773351 10.1002/ana.24335 PMC4737223

[69] L. Pavelka , A. Rauschenberger , Z. Landoulsi , et al., “Age at Onset as Stratifier in Idiopathic Parkinson's Disease – Effect of Ageing and Polygenic Risk Score on Clinical Phenotypes,” npj Parkinson's Disease 8, no. 1 (2022): 1–10. 10.1038/s41531-022-00342-7 PMC9363416 35945230

[70] M.‐W. Sia , J.‐N. Foo , S.‐E. Saffari , et al., “Polygenic Risk Scores in a Prospective Parkinson's Disease Cohort,” Movement Disorders 36, no. 12 (2021): 2936–2940. 34402545 10.1002/mds.28761 PMC8688232

[71] H. Leonard , C. Blauwendraat , L. Krohn , et al., “Genetic Variability and Potential Effects on Clinical Trial Outcomes: Perspectives in Parkinson's Disease,” Journal of Medical Genetics 57, no. 5 (2020): 331–338. 31784483 10.1136/jmedgenet-2019-106283 PMC8474559

[72] C. Blauwendraat , N. Tayebi , E. G. Woo , et al., “Polygenic Parkinson's Disease Genetic Risk Score as Risk Modifier of Parkinsonism in Gaucher Disease,” Movement Disorders 38, no. 5 (2023): 899–903. 36869417 10.1002/mds.29342 PMC10271962

[73] E. R. Dorsey , K. C. Darwin , S. Mohammed , et al., “Virtual Research Visits and Direct‐To‐Consumer Genetic Testing in Parkinson's Disease,” Digital Health 1 (2015): 2055207615592998. 29942542 10.1177/2055207615592998 PMC5999055

[74] T. L. Myers , C. G. Tarolli , J. L. Adams , et al., “Video‐Based Parkinson's Disease Assessments in a Nationwide Cohort of Fox Insight Participants,” Clinical Parkinsonism & Related Disorders 4 (2021): 100094. 34316671 10.1016/j.prdoa.2021.100094 PMC8299965

[75] A. R. Winslow , C. L. Hyde , J. B. Wilk , et al., “Self‐Report Data as a Tool for Subtype Identification in Genetically‐Defined Parkinson's Disease,” Scientific Reports 8, no. 1 (2018): 12992. 30154511 10.1038/s41598-018-30843-6 PMC6113219

